# Performance of Ultra-Deep Pyrosequencing in Analysis of HIV-1 *pol* Gene Variation

**DOI:** 10.1371/journal.pone.0022741

**Published:** 2011-07-25

**Authors:** Mattias Mild, Charlotte Hedskog, Johanna Jernberg, Jan Albert

**Affiliations:** 1 Department of Microbiology, Tumor and Cell Biology, Karolinska Institutet, Stockholm, Sweden; 2 Clinical Microbiology, Karolinska University Hospital, Stockholm, Sweden; University of Sao Paulo, Brazil

## Abstract

**Introduction:**

Ultra-deep pyrosequencing (UDPS) has been used to detect minority variants within HIV-1 populations. Some aspects of the quality and reproducibility of UDPS have been previously evaluated, but comprehensive studies are still needed.

**Principal Finding:**

In this study the UDPS technology (FLX platform) was evaluated by analyzing a 120 base pair fragment of the HIV-1 *pol* gene from plasma samples from two patients and artificial mixtures of molecular clones. UDPS was performed using an optimized experimental protocol and an in-house data cleaning strategy. Nine samples and mixtures were analyzed and the average number of reads per sample was 19,404 (range 8,858–26,846). The two patient plasma samples were analyzed twice and quantification of viral variants was found to be highly repeatable for variants representing >0.27% of the virus population, whereas some variants representing 0.11–0.27% were detected in only one of the two UDPS runs. Bland-Altman analysis showed that a repeated measurement would have a 95% likelihood to lie approximately within ±0.5 log_10_ of the initial estimate. A similar level of agreement was observed for variant frequency estimates in forward vs. reverse sequencing direction, but here the agreement was higher for common variants than for rare variants. UDPS following PCR amplification with alternative primers indicated that some variants may be incorrectly quantified due to primer-related selective amplification. Finally, the *in vitro* recombination rate during PCR was evaluated using artificial mixtures of clones and was found to be low. The most abundant *in vitro* recombinant represented 0.25% of all UDPS reads.

**Conclusion:**

This study demonstrates that this UDPS protocol results in low experimental noise and high repeatability, which is relevant for future research and clinical use of the UDPS technology. The low rate of *in vitro* recombination suggests that this UDPS system can be used to study genetic variants and mutational linkage.

## Introduction

In 2005 the first next generation sequencing (NGS) platform, the Genome Sequencer 20 (GS20) from 454 Life Sciences [1], was released and since then several other NGS platforms have been introduced. In addition, several updates have been released, which have increased the throughput and read length. The major platforms used today include the 454 FLX and 454 Titanium from Roche, the SOLID platform from Applied Biosystems and the Solexa platform from Illumina. SOLID and Solexa generate reads which are 50–100 base pairs (bp) long. The 454 FLX and Titanium platforms produce longer reads (300 and 500 bp, respectively). One application of NGS is sequencing of many individual template molecules obtained from specific gene fragments to study minority sequence variants and haplotype composition. For this approach the amplicon sequencing on the 454 platform, also called ultra-deep pyrosequencing (UDPS), is well suited because of the longer read lengths. The UDPS technology has been used to study cancer-associated genes in humans [2], [3] and minority variants within the population of human immunodeficiency virus type 1 (HIV-1), including drug resistance [4], [5], [6], [7], [8], [9], coreceptor use [10], [11], [12] and coevolution in the *nef* gene [13]. The sequence depth of the UDPS technology is limited by the experimental error and the number of input template molecules. Experimental errors may be introduced during the sequencing procedure [3] as well as the preceding reverse transcription and PCR amplification [9]. The PCR amplification is known to sometimes generate: 1) Artifactual substitutions, insertions and deletions: 2) Primer mismatches that may result in selective amplification failure of some sequence variants (this is especially relevant for HIV-1 and other targets with high genetic variability [14] and we will refer to this problem as primer-related selective amplification): and 3) *In vitro* recombination during PCR amplification [15], [16], [17], [18], [19] that may disrupt mutational linkage and thereby hinder studies of sequence variants. We have previously shown that the *in vitro* recombination frequency during UDPS is low [9], but here we extend these findings. Bioinformatic approaches have been developed to distinguish high confidence variants from sequencing artifacts. These approaches have been reported to decrease the sequencing error rate to levels ranging from 0.05% [9], [20] to 0.43% [11]. The error rate is not uniform across sites, but rather is higher in or adjacent to homopolymer tracts [3]. Hence, we have suggested that site-specific error rates should be used in studies of specific mutations [9]. The repeatability of HIV-1 variant frequency estimations using UDPS has been studied by Poon *et al.* [13], who analyzed three patients' plasma samples and showed that the repeatability was high for variants representing more than 1–5% of the virus population. Known variants representing 1% [12] and 0.1% [20] have been shown to be detectable by UDPS. Comparison of UDPS data in forward and reverse direction might facilitate data cleaning, but this has not been evaluated previously.

In this study, we have amplified a region of the HIV-1 *pol* gene of patients' plasma samples and molecular clones to evaluate the UDPS technology (FLX platform) for experimental noise and data variability, such as repeatability, effects of sequence direction, sensitivity, influence of primer-related selective amplification and *in vitro* PCR recombination.

## Materials and Methods

### Ethics statement

A research ethics application was approved by Regional Ethical Review Board in Stockholm, Sweden (Dnr 52/2008-77). The patients gave written informed consent according to the Declaration of Helsinki.

### Samples

**Figure 1:**
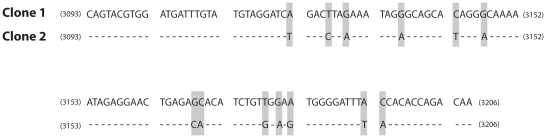
Nucleotide sequence alignment of clone 1 and clone 2. The clones cover position 3093–3206 in HXB2 and were used for the mixing experiments. The clones differed by 13 informative sites that are highlighted in gray.

For this study, we used four HIV-1 patient plasma samples (samples A, B, C and D). Sample A and B were used to study repeatability, effects of sequence direction and influence of primer-related selective amplification. These samples had approximately 1,050,000 and 1,600,000 HIV-1 RNA copies/ml, respectively. Plasma samples C and D were used to generate two molecular clones (clone 1 and clone 2) for studies on UDPS sensitivity and *in vitro* PCR recombination. These two clones were chosen on the basis of sequence dissimilarity with the aim to maximize the number of informative sites in the *pol* amplicon of interest (Figure 1).

### Generation of molecular clones

A 1320 base pair fragment of the HIV-1 *pol* gene was amplified with FastStart High Fidelity System (Roche, Penzberg, Germany) using the primers JA269 and JA272 [9]. The amplicon was cloned into a TOPO-TA cloning vector (Invitrogen, Carlsbad, California, US) and chemically competent TOP10 cells were transformed using heat. White colonies were picked and PCR amplified using JA269 and JA272 and sequenced with Sanger sequencing using Big Dye termination kit 3.1 (Applied Biosystems, Foster City, California, US) according to the manufacturer's instructions.

### Ultra-deep pyrosequencing (UDPS)

We performed PCR amplification and UDPS on a 167 base pair (bp) fragment of the HIV-1 *pol* gene as previously described [9]. The data analysis was focused on a 120 bp fragment within the amplicon, which corresponds to amino acid positions 180 to 219 of the reverse transcriptase (RT). Briefly, viral RNA was extracted from 200 µl plasma using RNeasy Lipid Tissue Mini Kit (Qiagen, Hilden, Germany) and [21] the QIAvac 24 vacuum manifold (Qiagen, Hilden, Germany). cDNA synthesis was done using primer JA272. The number of HIV-1 cDNA viral templates subjected to UDPS was quantified using an in-house limiting dilution PCR method [9].

Nested PCR was performed with outer primers JA269+JA272 followed by inner primers JA329+JA331 as previously described [9]. The 5′-ends of the forward and reverse inner primers included the specific UDPS adaptors A and B, respectively, as well as 4-nucleotide sample-specific tags, which were used to separate sequence reads from different samples. The PCR amplicons were purified using the GE PCR purification kit (GE health care, Pollards Wood, United Kingdom) and the DNA concentrations and purity were determined using Nanodrop (Thermo Fisher Scientific, Waltham, US) and the Agilent 2100 Bioanalyzer (Agilent Life Science, Santa Clara, California, US).

After quality controls, the PCR amplicons were sequenced in both forward and reverse direction on the 454 Life Science platform (GS-FLX, Roche Applied Science) according to the manufacturer's instructions. The two physical fields on the Picotiter plate were used and eight samples were mixed and analyzed in each field. We aimed at obtaining approximately 20,000 sequence reads from each sample. After UDPS, the read from different samples were identified by in-house scripts and the sample-specific sequence tags. The characteristics of the samples and basic information about the UDPS are summarized in Table 1.

**Table 1:** Characteristics of the samples and basic information on UDPS.

Experiment	Sample and run characteristics	Approx. no. of templates	No. of UDPS reads after data cleaning
Repeatability	Plasma A, run 1	40,000	26,846
	Plasma A, run 2	40,000	23,376
	Plasma B, run 1	10,000	14,614
	Plasma B, run 2	10,000	11,934
Sensitivity	Clone mix 0.5∶99.5	30,000	23,668
	Clone mix 0.05∶99.95	30,000	25,622
Primer-related selective amplification	Plasma B – alt. primers	10,000	8,858
*In vitro* recombination	Clone mix 50∶50	100,000	20,469
	Clone mix 50∶50	10,000	19,245

### Repeatability of variant quantification

The plasma samples A and B were analyzed two times each. The RNA extraction, cDNA synthesis and PCR were performed on two separate time points and sequenced in the same UDPS run [9]. The number of input cDNA templates for sample A and B were approximately 40.000 and 10.000, respectively.

### Sensitivity of detecting minority variants

Clone 1 and clone 2 were used to generate two mixtures with 99.5∶0.5 and 99.95∶0.05 ratios. The two mixtures were used for PCR amplification and UDPS. For both experiments we used approximately 30,000 templates (determined using Nanodrop) as input in the outer PCR reaction. We aimed at obtaining a total of 20,000 reads per sample. Thus, we attempted to detect 100 and 10 molecules, respectively, of the minor variant against a background of approximately 20,000 molecules of the major variant.

### Primer-related selective amplification

To determine the possible effect of selective amplification of certain variants due to primer-related selective amplification, plasma sample B was re-extracted and re-analyzed with an alternative set of nested PCR primers, JA270+JA271 and JA323+JA332, which amplify a 316-bp fragment that completely encompasses the 167-bp region targeted by the first primer set. None of the alternative primers overlapped with the original primers. Both primer sets were designed to hybridize to conserved regions of the HIV-1 *pol* gene and included wobbled bases to further minimize the risk of selective amplification of certain viral variants. We performed cDNA synthesis with JA271, CCACTAAYTTCTGTATRTCATTGAC (position 3309–3334 in HXB2) and a nested PCR with outer primers JA271+JA270, GCTTCCCTCARATCACTCTTA (position 2248–2268 in HXB2), and inner primers JA323, TGGAAAGGATCACCAGCRATA (position 3006–3026 in HXB2) and JA332, GCTGTACTGTCCATTTRTCAGGATG (position 3276-3252 in HXB2). The inner primers contained the same UDPS adaptors and sample tags sequences as described above. Both the outer and inner PCR cycling profiles were as follows: 94°C for 2 min, 30 cycles of 94°C for 20 sec, 50°C for 20 sec and 72°C for 1 min 30 sec, followed by a final extension at 72°C for 6 min and finally at 4°C until used. The input number of templates for UDPS was approximately 10,000 molecules.

### Evaluation of in vitro PCR recombination

Clone 1 and clone 2 were mixed in a 50∶50 ratio before PCR amplification and UDPS. The clones differed by 13 informative sites. We analyzed two mixtures with 100,000 and 10,000 DNA templates (determined using Nanodrop, Thermo Fisher Scientific, Waltham, US), respectively, as input in the outer PCR. A recombinant was defined as a sequence that had replacement of at least two signature nucleotides irrespective of whether they were adjacent or not (Figure 1).

### Data cleaning

The data cleaning was performed using in-house filtering scripts (Jernberg et al., manuscript in preparation) as outlined previously [9] and in Table S1. The data cleaning was designed to remove reads with probable sequencing errors and was based on UDPS analyses of an HIV clone. The filters used for each experiment and the number of reads retained in each step are shown in Table S1. Briefly, the scripts filter: 1) All reads with low similarity to the corresponding Sanger sequence (in this study we used an 80% similarity cut-off). 2) Reads that did not cover the entire region of interest (amino acids 180–219 in RT). 3) Reads containing ambiguous bases (Ns). Remaining reads were imported into the GS amplicon software (Roche, Penzberg, Germany) and aligned. 4) The alignment was extracted and cut to the region of interest (amino acid 180–219). The sequence data were compressed by scripts that identified unique sequence variants in forward and reverse direction and counted the number of reads per variant. Additional scripts filtered: 5) Reads with out-of-frame indels or stop codons while retaining reads with in-frame indels (i.e. ±3, 6, 9 nucleotides). 6) The alignments were manually inspected and any remaining variants with frameshifts or stop codons were removed. 7) In the experiments on patient plasma samples we used a previously defined cut-off value (0.11% [0.09–0.21%]) for detection of high-confidence variants [9]. Only the high-confidence variants that were detected in both forward and reverse direction were retained for further analysis. However, in the experiment where the influence of sequence direction was evaluated, we also assessed variants that only were observed in one sequence direction.

### Statistical analyses

We investigated repeatability of variant quantification using Bland-Altman analyses and plots [22]. The number of reads per variant was log transformed and the number of reads in the second (repeat) measurement was weighted by the number of reads in the first measurement. The Bland-Altman plot shows the average of each paired measurement on the x-axis and the difference between the paired measurements on the y-axis. The standard deviation (SD) of all the individual differences is calculated as a measure of repeatability. The limits of agreement are defined as the mean difference ±1.96 SD and represent the range within which approximately 95% of the differences will lie if they are normally distributed (which they were for our data following log transformation). We also calculated 95% confidence intervals (CI) of the limits of agreement according to Bland and Altman [22]. In addition, we calculated variance-to-mean ratios to allow comparisons with results published by Poon *et al* [13].

The difference between the number of recombinant variants in the 10,000 and 100,000 template experiments was compared using chi-square statistics. We compared the number of unique variants, rather than the total number of recombinant reads, since each recombinant will be PCR amplified and therefore may appear in one or several reads depending on in which PCR cycle they were generated.

## Results

The data cleaning was performed in a hierarchical manner by an in-house method (Jernberg et al., manuscript in preparation) as outlined previously [9]. During this process we discarded on average 20% (range 8–34%) of the reads per sample. Detailed information on the cleaning procedure and the number of reads retained after each cleaning step is shown in Table S1.

### High repeatability of variant quantification

To evaluate the repeatability of frequency estimates of HIV-1 variants using UDPS, we repeated the complete experimental protocol for two patient plasma samples (sample A and sample B). Thus, these experiments evaluated the repeatability of the RNA extraction, cDNA synthesis and PCR. The analyses of repeatability were done on “high-confidence” variants, which had been identified using a data cleaning procedure that removed probable sequencing artifacts (see Materials and Methods and [9]). For sample A, a total of 27 variants were detected in both run A∶1 and run A∶2. The least abundant variant that was detected in both runs represented on average 0.11% of the viral population (Table 2). In addition, there were six high-confidence variants that were unique to run A∶1, i.e. not detected in run A∶2. These unique variants represented between 0.11 and 0.23% of the population. Similarly, there were four unique variants in run A∶2 that represented between 0.12% and 0.13% of the population. In sample B, 15 variants were identified in both run B∶1 and run B∶2 and the least abundant variant represented on average 0.17% of the population (Table 2). In addition, eight unique variants were found in run B∶1 (representing between 0.15 and 0.27%) and seven unique variants were found in run B∶2 (representing between 0.12 and 0.21%).

**Table 2:** Limit of detection of repeatedly detected virus variants in samples analyzed using original primers and alternative PCR primers.

Sample	No. of variants detected in both runs	Lowest proportion (%) of variants detected in both runs (run 1, run 2)	Highest proportion (%) of variants detected in only one run
Plasma A, run 1	27	0.11 (0.11, 0.11)	0.230.13
Plasma A, run 2			
Plasma B, run 1	15	0.17 (0.18, 0.16)	0.270.21
Plasma B, run 2			
Plasma B, run 1	14	0.19 (0.16, 0.21)	0.400.34
Plasma B – alt. primers			

**Figure 2:**
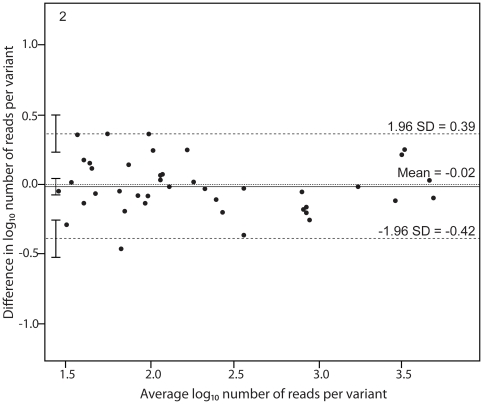
Bland-Altman plot showing the repeatability of variant quantification using UDPS. UDPS was performed twice for sample A and sample B. These paired measurements were combined and the number of reads in the second (repeat) measurement was weighted by the number of reads in the first measurement. The number of reads per variant was log transformed. The differences in number of reads per variant in the repeat analyses are plotted against the average number of reads per variant. Horizontal lines are drawn at the mean difference between the two measurements and at the upper and lower limits of agreement. The 95% confidence intervals are also shown for the mean and the upper and lower limits of agreement.

Figure 2 shows a Bland-Altman plot of the repeatability of quantification of virus variants in sample A and B [22]. Individual Bland-Altman plots of sample A and sample B gave similar results (data not shown). The mean log_10_ difference between the two measurements was −0.02 (95% CI: −0.08–0.05). The upper limit of agreement was 0.39 (95% CI: 0.22–0.50) and the lower limit of agreement was −0.42 (95% CI: −0.53–−0.31). This means that a repeated measurement would have a 95% likelihood to lie approximately within a factor ±0.5 log_10_ of the initial estimate. Thus, a variant that was found in 100 reads in the first measurement had a 95% likelihood to lie between 32 and 320 reads in the second measurement. Somewhat unexpectedly there was no relationship between the repeatability of quantification and the abundance of the variants (Spearman R = −0.054, p = 0.73).

In a previous publication Poon *et al.* [13] investigated UDPS repeatability by calculations of the variance-to-mean ratios. To allow comparison with these results we did similar calculations. The variance-to-mean ratio for sample A ranged from 2.5×10^−2^ to 4.7×10^−7^ and had a median value of 1.1×10^−4^. For sample B, the variance-to-mean ratios ranged from 5.9×10^−2^ to 6.7×10^−6^, with a median value of 8.2×10^−4^. The average variance-to-mean ratio in the two experiments was 3.2×10^−4^.

**Figure 3:**
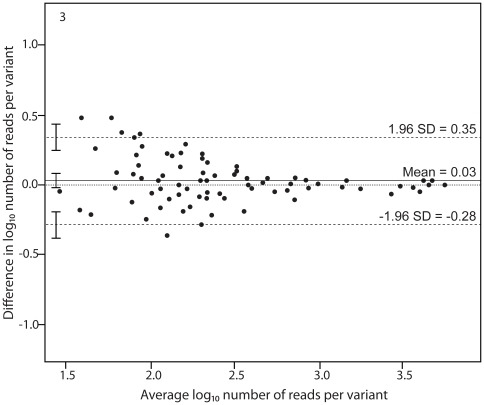
Bland-Altman plot showing the agreement of variant quantification in forward and reverse direction. The data from the paired measurements from sample A and sample B were combined and the number of reads in the reverse direction was weighted by the number of reads in the forward direction. The number of reads per variant was log transformed. The differences in number of reads per variant in forward and reverse direction are plotted against the average number of reads per variant. Horizontal lines are drawn at the mean difference between the two measurements and at the upper and lower limits of agreement. The 95% confidence intervals are also shown for the mean and the upper and lower limits of agreement.

We also investigated the agreement of quantification in forward vs. reverse reads for the 27 and 15 variants observed in sample A and sample B, respectively. A Bland-Altman analysis of the combined data from the repeated measurement of sample A and sample B showed that the mean log_10_ agreement between the forward and reverse measurements was 0.03 (95% CI: −0.01–0.08) (Figure 3). The upper limit of agreement was 0.35 (95% CI: 0.26–0.43) and the lower limit of agreement was −0.28 (95% CI: −0.36–−0.20). This is similar to the repeatability in the re-analysis experiments described above. However, in contrast to these experiments, the agreement between forward and reverse analyses was higher for common variants than for rare variants (Spearman R = 0.63, p<0.001). We also studied variants that were found only in one direction (forward or reverse). Together these variants represented on average 3.8% (range 2.0 to 4.9%) of the total amount of reads in each direction, but all such variants were rare and constituted between 0.07 and 0.19% of the virus population (Table S2).

Collectively these results showed that we were able to detect viral variants that represented down to 0.11% of the virus population. The repeatability was good for both major and minor variants. Thus, the experimental noise introduced during the RNA extraction, cDNA synthesis and PCR was low. However, there was a stochastic effect that primarily affected the ability to consistently detect rare variants.

### Minority variants can be detected

The error frequency of our experimental system has previously been shown to be 0.11% (range 0.09–0.21%) at the variant level [9], which means that theoretically it should be possible to detect a single molecule of one variant against a background of approximately 1000 molecules representing other variants. In line with this the repeated UDPS analyses of plasma samples A and B above indicated that minority variants constituting >0.27% could be detected and reproducibly quantified. To further investigate the lower limit of detection of viral variants we performed UDPS on two molecular clones that had been mixed at ratios of 99.5∶0.5 and 99.95∶0.05. In the first experiment (ratio 99.5∶0.5) we obtained 23,668 reads of which 524 (2.2%) were the minority variant. In addition, 21 recombinant reads were identified, corresponding to 0.089%. In the second experiment (ratio 99.95∶0.05), 79 (0.31%) of the total 25,622 reads were found to be the minority variant. Here, we only identified a single recombinant sequence read, representing 0.0039% of the total population. These data suggest that minor HIV-1 variants that constitute as little as 0.05% of the viral population can be detected by UDPS, but we cannot rule out the possibility that our artificial mixtures contained slightly higher proportions of the minor virus variant than intended.

### Potential selective PCR amplification as a result of primer mismatch

**Figure 4:**
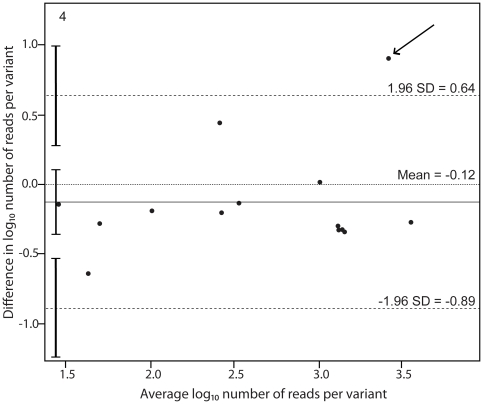
Bland-Altman plot showing the agreement of variant quantification using two primer sets. Templates for UDPS were prepared using original and alternative PCR primers. The number of reads in the second (alternative primers) measurement was weighted by the number of reads in the first measurement. The number of reads per variant was log transformed. The differences in number of reads per variant using the two primer sets are plotted against the average number of reads per variant. Horizontal lines are drawn at the mean difference between the two measurements and at the upper and lower limits of agreement. The 95% confidence intervals are also shown for the mean and the upper and lower limits of agreement.

The effect of primer design was evaluated by re-extracting sample B and performing cDNA and PCR with an alternative set of nested primers. Fourteen high-confidence variants were found in both experiments and the least abundant variant represented 0.19% of the population (Table 2). In addition, 12 variants (representing between 0.15 and 0.40%) were only found when the original primer set was used and eight variants (representing between 0.12 and 0.34%) were only found when the alternative primer set was used. Figure 4 shows a Bland-Altman plot of the agreement of variant frequency estimates using the two primer sets. The mean log_10_ difference between the forward and reverse measurements was −0.12 (95% CI: −0.34–0.09). The upper limit of agreement was 0.64 (95% CI: 0.26–1.01) and the lower limit of agreement was −0.89 (95% CI: −0.51–−1.26). Thus, the limits of agreement were approximately two times wider than when UDPS was repeated with the same primers. The main reason for this difference was that a single variant, which represented 46% of the virus population in the analyses with the original primers, only represented 5.6% in the analysis with the alternative primers (outlier marked by an arrow in Figure 4). This suggests that this particular variant was selectively under-quantified by the alternative primers, presumably due to a primer mismatch problem. Since it was a major variant the estimates of the proportions of all other variants were also affected. Accordingly, the agreement between the analyses with the original and alternative primers was higher if the problematic variant was omitted (data not shown).

### Low level of in vitro recombination

UDPS has been used to study genetic variants and mutational linkage, but such analyses are only valid if the frequency of *in vitro* recombination is zero or low. To determine the *in vitro* recombination frequency in our experimental system we mixed two molecular clones in a 50∶50 ratio before PCR amplification and UDPS. The two clones differed by 13 informative sites that were distributed over the fragment (Figure 1). In addition, to study if the frequency of *in vitro* PCR recombination may be influenced by the number of target molecules we tested both 100,000 and 10,000 HIV DNA templates as input in the outer PCR.

**Figure 5:**
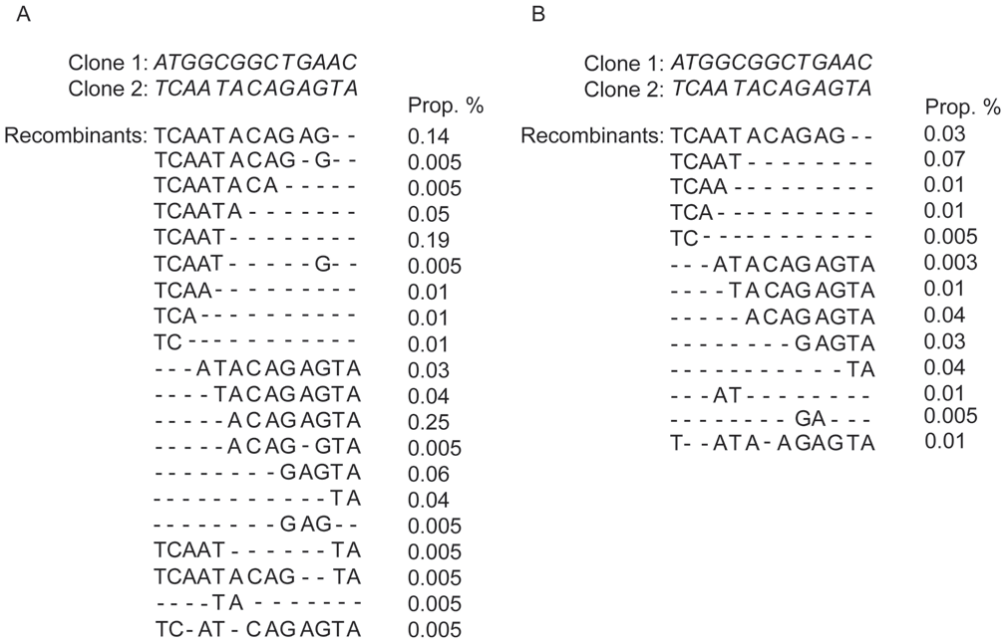
Analysis of *in vitro* PCR recombination. The informative sites of clone 1 and clone 2 (see Figure 1) are shown in italics in the upper part of the figure. Below are identified recombinant reads presented for the experiment with 100,000 input template molecules (panel A) and 10,000 input template molecules (panel B). The proportion (Prop. %) of each recombinant is also shown.

In the experiment where 100,000 input HIV DNA templates were used, we identified 182 recombinant reads among a total 20,469 reads, which corresponds to an *in vitro* recombination frequency of 0.89%. Based on the signature nucleotides, the recombinant reads consisted of 12 single recombinants, which represented between 0.005 and 0.25% of all reads, and four double recombinants, which represented 0.005% each (Figure 5A). When 10,000 templates were used as input we found 56 recombinant reads among a total 19,245 reads, which corresponds to a recombination frequency of 0.29%. There were 10 single recombinants (representing between 0.005 and 0.07%) and two double recombinants (representing 0.01% and 0.005%) (Figure 5B). In the experiment with 100,000 and 10,000 input molecules, we identified a few possible triple and quadruple recombinants ranging from 0.005 to 0.01% (Figure 5A and Figure 5B). However, it is difficult to determine if these reads have been generated by recombination, substitution or a combination of both. The difference between the numbers of recombinant variants in the 100,000 template experiment compared the 10,000 template experiment was not statistically significant (p = 0.47, chi-square test). Taken together, these results showed that the *in vitro* recombination frequency was low in our experimental system, which allows us to study mutational linkage and identify genetic variants.

## Discussion

In this study we have evaluated the sensitivity, repeatability, primer-related selective amplification and *in vitro* PCR recombination of a UDPS protocol that targets a 120 base pair fragment of the HIV-1 *pol* gene. We found that our system was capable of delivering repeatable results for variants representing >0.27% of the population. The repeatability of quantification of viral variants was approximately ±0.5 log. A similar degree of agreement was observed between forward and reverse reads. Furthermore, our results indicate that the choice of primers may be important when analyzing highly variable sequences, like HIV-1, due to the risk of primer-related selective amplification. Finally, the *in vitro* recombination rate during PCR was low, suggesting that our UDPS method can be used to study genetic variants and mutational linkage.

To evaluate the repeatability of frequency estimates of HIV variants, we performed repeated UDPS analyses of two patient plasma samples. We found that a repeated measurement would have a 95% likelihood of lying within ±0.5log_10_ of the initial estimate. Interestingly, the repeatability was similar for rare and more abundant variants. We also compared our results with those of Poon *et al*. [13], who used variance-to-mean ratios to investigate repeatability. The average variance-to-mean ratio in our experiments was 3.2×10^−4^, which is more than 20 times lower than that estimated by Poon *et al.* [13]. In addition, they reported that some variants that represented 1–5% of the virus population in one analysis were not detected when the analysis was repeated. Similarly, Gianella *et al*. recently reported a low level of repeatability in detection and quantification of minority drug resistance mutations [23]. We repeatedly identified all variants that represented >0.27% of the virus population. The reason for the differences in repeatability between these studies and ours is not clear, but could be due to differences in both laboratory methodology, sequencing approach and data cleaning. For instance Gianella *et al*. used a shotgun sequencing approach, which generally gives lower sequence depth (coverage) than amplicon sequencing. In summary, we found that our system had good repeatability, which indicates that the experimental noise introduced during RNA extraction, cDNA synthesis, PCR and UDPS was low. However, and as illustrated above, the performance of our UDPS system cannot be directly translated to other UDPS systems, since both the error rate and the reproducibility depends on many factors such as experimental methodology, amplicon length, UDPS platform and data cleaning strategy. Furthermore, our cleaning strategy has been optimized for this amplicon, but the filters and their settings can be changed by the user to obtain a less stringent data cleaning if desired. In fact, we recommend that each step in the cleaning process should be optimized for each gene region analyzed and according to the purpose of the analyses.

Analysis of bidirectional UDPS has been described in only a few studies [9], [11], [24], in which variants were considered “true” if they were present in both sequence directions. Here, we have studied the effect of sequence direction on variant abundance estimates. We found that the difference in variant abundance between forward and reverse sequence direction was relatively small and approximately as great as the difference between UDPS runs. However, in contrast to these experiments, the agreement between forward and reverse analyses was higher for common variants than for rare variants. In addition, some variants only exceeded our cut-offs for high-confidence variants in one sequence direction. These variants would be considered “true” if sequencing was done in only one direction (forward or reverse) or if the requirement that a variant need to be present in both forward and reverse direction would be ignored. It is not surprising that there is some stochasticity in the ability to detect rare variants that have an abundance that is close to the detection limit.

We tested the ability of our UDPS methodology to identify minority variants representing 0.5 and 0.05% of the population using mixing experiments of molecular clones. The minor variant was identified in both experiments, but the proportions were somewhat higher than intended, i.e. 2.2% and 0.31% respectively. This may be a stochastic effect, but we cannot exclude the possibility that minority strains may have been systematically overestimated for instance if major variants have reached the PCR plateau earlier than rare variants. Artificial HIV-1 mixtures of 1% and 0.1% have been analyzed by Tsibris *et al.* [12] and Zagodi *et al.* [20], respectively. Our results are in agreement with those by Tsibris *et al.* and Zagordi *et al.*, and suggest that it is possible to detect minor variants of the HIV-1 population, at least when the minor variant is genetically clearly distinguishable from the major variants such as in the case of superinfections.

We also evaluated the potential influence of primer-related selective amplification on estimation of variant abundance using alternative primer sets that targeted the same region in the *pol* gene. Despite our efforts to design two optimal sets of nested primers that targeted highly conserved primer binding sites and included wobbled nucleotides, the estimations of variant abundance differed between the two primer sets. We were able to detect variants down to 0.2% of the viral population with both primer sets. However, one variant, which was estimated to represent 46% using the original primers, was detected in only 5.6% of the reads obtained with the alternative primers. As a result the limits of agreement was approximately two times wider than when the sample was re-analyzed with the original primer set. This suggests differential amplification of certain HIV-1 variants, presumably due to primer- related selective amplification. Thus, optimal primer design may be very important when UDPS is used to analyze the population structure in divergent target sequences, like HIV-1 populations. One could even speculate if multiple primer sets should be used in order to fully and correctly characterize HIV-1 variation.

We found that the frequency of *in vitro* recombination was 0.89% and 0.29% when 100,000 and 10,000 templates were used as input, respectively. Most recombinants were represented by a very low number of reads and most of these variants would be removed by our data cleaning strategy since their abundances were lower than our cut-off for high-confidence variants (0.11%) [9]. The frequency of *in vitro* recombination that we estimated was higher than reported by Tsibris et al. [12] (0.11 to 0.15%) but lower than that reported by Zagordi *et al*. [20](1.9%). However, while we used 50∶50 clonal mixtures, Tsibris *et al.* used an 89∶10∶1 mixture and the likelihood of *in vitro* recombination during PCR should be higher with 50∶50 mixtures. The higher *in vitro* recombination frequency reported by Zagordi *et al.*, who used a mixture of 10 clones in proportions of 0.3 to 30%, is probably due to a longer amplicon, but could also be due to differences in laboratory methodology or data cleaning strategies. It is likely that most *in vitro* recombinants are generated during PCR and consequently *in vitro* recombination frequency will probably increase if larger amplicons are analyzed. This is relevant when longer amplicons are analyzed using the Titanium platform that can analyze up to 500 bp long fragments and future platforms that will be able to analyze even longer fragments. However, it may be possible to reduce PCR-induced recombination by lowering the cycle number, increasing the extension time and decreasing the initial template concentration [16], [17], [19], [20]. Furthermore, the choice of DNA polymerase may be of importance [20]. Here, we have shown that *in vitro* recombination is low for our PCR methodology. However, we found individual recombinants representing up to 0.25% of the population, which implies that *in vitro* recombination cannot be excluded for rare variants.

One limitation of this study should be recognized. The number of samples included in the study was limited and some of the experiments were not repeated. However, we believe the results show the capacity of our UDPS system and the results also highlight the importance of including control experiments in UDPS studies.

In conclusion, we have performed a series of experiments to evaluate the performance of UDPS analysis of a region of the HIV-1 *pol* gene. The results show that the repeatability was good for major as well as minor variants in patient plasma samples. For rare variants *in vitro* recombination and effects of sequence direction needs to be considered. Finally, the design of primers for PCR amplification is of special importance during UDPS, since primer-related selective amplification can skew frequency estimates of genetic variants. The results are of relevance for future research and clinical use of the UDPS technology.

## Supporting Information

Table S1 The number of total reads and the number of reads retained per sample as a percent of raw reads.
(DOC)

Table S2 Repeatability of frequency estimates in forward and reverse direction.
(DOC)
