# Supplementary material for: Performance of Ultra-Deep Pyrosequencing in Analysis of HIV-1 pol Gene Variation
Source: PLoS One. 2011 Jul 25;6(7):e22741. doi: 10.1371/journal.pone.0022741 (PMC3143174; doi:10.1371/journal.pone.0022741)
Supplement: Table S1 — The number of total reads and the number of reads retained per sample as a percent of raw reads. (DOC) [file pone.0022741.s001.doc]

**Supplementary table 1.** The number of total reads and the number of reads retained per sample as a percent of raw reads.

|  |  |  |  | **Cleaning step**  **(Number of reads remaining / reads remaining in %)** | | | | | | |
| --- | --- | --- | --- | --- | --- | --- | --- | --- | --- | --- |
| **Experiment** | **Sample** | **Sequence direction** | **Raw reads** | **80% similarity** | **Read length** | **Ambiguous bases (N’s)** | **Out-of-frame indels** | **Stop codons** | **Manual removal of indels** | **High confidence variants** |
| **Repeatability**  **Forward vs. Reverse** | A:1 | Forward | 12320 | 12274  / 99.6% | 9695  / 78.7% | 9347  / 75.9% | 8421  / 68.3% | 8327  / 67.6% | 8288  / 67.3% | - |
|  |  | Reverse | 26221 | 26129  /99.6% | 24206  / 92.3% | 23824  / 90.9% | 21930  / 83.6% | 21695  / 82.7% | 21592  / 82.3% | - |
|  | A:2 | Forward | 11394 | 11350  / 99.6% | 8799  / 77.2% | 8503  / 74.6 % | 7627  / 66.9% | 7520  / 66.0% | 7477  / 65.6% | - |
|  |  | Reverse | 23223 | 23177  / 99.8% | 21566  / 92.8% | 21232  / 91.4% | 19007  / 81.8% | 18722  / 80.6% | 18549  / 79.9% | - |
|  | B:1 | Forward | 10291 | 10287  / 100% | 10199  / 99.1% | 9987  / 97.0% | 9514  / 92.4% | 9492  / 92.2% | 9482  / 92.1% | - |
|  |  | Reverse | 7381 | 7363  / 99.8% | 7303  / 98.9% | 7163  / 97.0% | 6825  / 92.5% | 6799  / 92.1% | 6797  / 92.1% | - |
|  | B:2 | Forward | 9170 | 9154  / 99.8% | 8039  / 87.7% | 7815  / 85.2% | 7352  / 80.2% | 7321  / 79.8% | 7309  / 79.7% | - |
|  |  | Reverse | 7049 | 7030  / 99.7% | 6531  / 92.6% | 6399  / 90.8% | 6077  / 86.2% | 6038  / 85.7% | 6035  /85.6% | - |
| **Repeatability** | A:1 | Forward + Reverse | 38541 | s | s | s | s | 29629 ^1^  / 76.9% | 29582  / 76.7% | 26846  / 69.7% |
|  | A:2 | Forward + Reverse | 34617 | s | s | s | s | 25918 ^1^  / 74.9% | 25834  / 74.6% | 23376  / 67.5% |
|  | B:1 | Forward + Reverse | 17672 | s | s | s | s | 16195 ^1^  / 91.6% | 16142  / 91.3% | 14614  / 82.7% |
|  | B:2 | Forward + Reverse | 16219 | s | s | s | s | 13169 ^1^  / 81.2% | 13163  / 81.2% | 11934  / 73.6% |
| **Sensitivity** | 0.5 : 99.5 | Forward | 12026 | - | - | 11592  / 96.4% | 10615  / 88,3% | # | 23668 ^2^  / 89.9% | - |
|  |  | Reverse | 14306 | - | - | 13809  / 96.5% | 13054  /91.2% | # |  | - |
|  | 0.05 : 99.95 | Forward | 13612 | - | - | 13318  / 97.8% | 12519  / 92.0% | # | 25622 ^2^  / 92.3% | - |
|  |  | Reverse | 14143 | - | - | 13907  / 98.3% | 13105  92.7% | # |  | - |
| **Primer selection** |  | Forward | 6456 | 6428  / 99.6% | 6004  / 93.0% | 5825  / 90.2% | 5319  / 82.3% | 9811 ^1^  / 81.8% | 9802  / 81.7% | 8858  / 73.8% |
|  |  | Reverse | 5539 | 5470  / 98.8% | 5206  / 94.0% | 4998  / 90.2% | 4624  / 83.4% |  |  |  |
| ***In vitro* recombination** | 100000 | Forward | 10522 | - | - | 10191  / 96.9% | 9400  / 89.3% | # | 20469 ^2^  / 90.3% | - |
|  |  | Reverse | 12158 | - | - | 11757  / 96.7% | 11073  / 91.1% | # |  | - |
|  | 10000 | Forward | 10127 | - | - | 9698  95.8% | 9068  / 89.5% | # | 19245 ^2^  / 91.1% | - |
|  |  | Reverse | 11003 | - | - | 10637  96.7% | 10125  / 92.0% | # |  | - |

The data cleaning was performed using in-house filtering scripts. The filters used and the number of reads retained in each step are shown; S: same as above except for that the forward and reverse reads were combined; - was not done; # for the clone experiments the stop codon (out-of frame) cleaning step was not performed since we wanted to study the reproducibility of these errors. ^1^ For patient plasma samples forward and reverse variants were combined and the abundance of the variants was calculated as described by Hedskog et al and if a variant was absent in one sequence direction it was discarded. ^2^ For the clone experiments the forward and reverse reads was set to the sum of the two estimates and variants only seen in one sequence direction were retained.
