# Supplementary material for: Performance of Ultra-Deep Pyrosequencing in Analysis of HIV-1 pol Gene Variation
Source: PLoS One. 2011 Jul 25;6(7):e22741. doi: 10.1371/journal.pone.0022741 (PMC3143174; doi:10.1371/journal.pone.0022741)
Supplement: Table S2 — Repeatability of frequency estimates in forward and reverse direction. (DOC) [file pone.0022741.s002.doc]

**Supplement table 2.** Repeatability of frequency estimates in forward and reverse direction

| **Sample** | **Prop. of unique**  **variants (%) ^1^** | | **Prop. of the most abundant unique variant (%) ^2^** | |
| --- | --- | --- | --- | --- |
|  | **Forward** | **Reverse** | **Forward** | **Reverse** |
| Plasma A, run 1 | 2.2 | 3.9 | 0.11 | 0.07 |
| Plasma A, run 2 | 2.0 | 4.5 | 0.11 | 0.08 |
| Plasma B, run 1 | 4.3 | 3.6 | 0.16 | 0.16 |
| Plasma B, run 2 | 4.9 | 4.6 | 0.19 | 0.10 |

^1^ Frequency estimates of unique variants found in only one sequence direction.

^2^ Proportion of the most abundant unique variant found in forward and reverse direction
